# Beyond fire: Flower production naturally occurs and is also influenced by leaf removal in a Neotropical savanna herb

**DOI:** 10.1371/journal.pone.0305098

**Published:** 2024-06-10

**Authors:** Heloisa S. Miranda, Pedro H. B. Togni, Ademar B. Dantas-Junior, Cassia B. R. Munhoz, Margarete N. Sato, Augusto C. Franco

**Affiliations:** 1 Department of Ecology, Institute of Biological Sciences, University of Brasília, Brasília, Distrito Federal, Brazil; 2 Graduate Program in Ecology, Institute of Biological Sciences, University of Brasília, Brasília, Distrito Federal, Brazil; 3 Department of Botany, Institute of Biological Sciences, University of Brasília, Brasília, Distrito Federal, Brazil; Makerere University, UGANDA

## Abstract

Several herbaceous species exhibit mass flowering after fires in Neotropical savannas. However, unequivocal evidence of fire dependency and the consequences for plant reproduction are lacking. In nutrient-poor fire-prone savannas, the damage caused by fire and by other means (e.g., leaf removal, but not necessarily having a negative impact) constrains the maintenance and expansion of plant population by affecting the ability of individuals to recover. Therefore, the compensatory responses of plants to both damages should be convergent in such environments. Using *Bulbostylis paradoxa*–reported to be fire-dependent to flower–as a model, we investigated the role of fire and leaf removal in anticipating the flowering and reproduction periods, and its possible consequences on seedling establishment. We monitored 70 burned individuals, 70 damaged/clipped, and 35 without damage to estimate time for flowering, seed quality and germination parameters. To expand our sampling coverage, we examined high-resolution images from herbarium collections in the SpeciesLink database. For each herbarium image, we recorded the presence or absence of a fire scar, the month of flowering, and the number of flowering stalks. *Bulbostylis paradoxa* was fire-stimulated but not dependent on fire to flower, with 65.7% of the individuals flowering in the burned area, 48.6% in the clipped, and 11.4% in the control. This was consistent with the analysis of the herbarium images in which 85.7% of the specimens with flowers had fire scars and 14.3% did not. Burned individuals synchronized flowering and produced more viable seeds. However, the seeds might face a period of unsuitable ecological conditions after early to mid-dry season fires. Flowering of unburned plants was synchronized with the onset of the rainy season. Flexibility in flowering and vegetative reproduction by fragmentation confer to this species, and most likely other plants from the herbaceous layer, the capability of site occupation and population persistence in burned and unburned savanna sites.

## Introduction

Stimulation of post-fire flowering has been associated with specific effects of fire, such as the removal of leaves and stems [1–3], plant size and age [4, 5], post-fire soil conditions (nutrient content, smoke exposure, humidity, and temperature), or a combination of factors (see [6]). However, in the absence of fire, one or more of these factors may also stimulate flowering, suggesting that the flowering is not fire dependent. In fact, Lamont & Downes [7] argue that an obligated fire-dependent species blooms only in the presence of fire, while a facultative fire stimulated species continues flowering indefinitely at a reduced rate, ensuring genetic variability, site occupation, and the species persistence at the community level.

Fire has been present in the terrestrial ecosystem for millions of years, acting as a selective filter for functional traits among species [8, 9]. Thick bark, buds protected by leaf sheaths, and the ability to resprout from belowground are plant traits previously associated to adaptations to fire [10, 11]. In the Cerrado, the Brazilian savanna, fires have occurred for at least 4 million years [12]. Nowadays, fires are mostly anthropogenic during the dry season, and are fast surface fires that typically consume ~94% of the fine fuel [13], top-killing most individuals of the herbaceous layer that, as in some fire-prone ecosystems, resprout shortly after the fire [14] and, in many cases, exhibit mass flowering [6, 7, 15, 16] within hours after the fire [17], a few weeks, or even 1 to 2 years later [6]. Fire stimulated flowering has been extensively reported for fire-prone ecosystems [6, 7, 18, 19]. For the Cerrado, the first reports of fire stimulated flowering were presented by the first European naturalists that visited Central Brazil in the 19^th^ century, and several studies reported mass post-fire flowering at species or community level [15–17, 20–22]. At the community level, circa 10% of the herbaceous species flower in the first 15 days after the fire event reaching 21.3% a month later [16] which gives them an advantage to resources use, decrease competition for pollinators, and for occupation of open spaces [18, 23].

The studies for Cerrado considered mostly the presence and absence of flowering species in burned and unburned areas, with few explicitly designed to investigate fire-dependence [1, 20, 24–26]. Although fire-stimulated flowering, either anticipating the flowering period or increasing the flower production, has been reported [15, 16, 26, 27], unequivocal evidence of fire-dependent flowering in species from the Cerrado is lacking. In the absence of fire, flowering of the herbaceous vegetation in Neotropical savannas occurs throughout the year and is strongly associated with rainfall [15, 28, 29]. However, the number of flowering individuals is significantly reduced with fire exclusion [15, 16, 21, 28], maintaining a low-density transient soil seed bank [30], that together with vegetative recruitment [14, 31], contribute to the persistence of herbaceous species in Cerrado communities. Clipping or removing the above-ground biomass also stimulates flowering at the individual or community level in the Cerrado but with a lower flower production than when stimulated by fire [1, 20, 21].

Recently Fidelis and Zirondi [32] and Zirondi *et al*. [16] reported that 12% to ~33% of the herbaceous layer species are fire-dependent to flower. These values are similar to the minimum values reported for fire-dependent species by Lamont and Downes [7] for Australasian and South Africa/Madagascar regions (28% to 44%). As fire is not the only factor affecting flowering on Neotropical species (see the examples above) and that fire dependency is not a widespread phenomenon in the Australasian and South Africa/Madagascar regions [7] and in the Cerrado [16, 32] raises the question whether fire is the only selective filter affecting the allocation of resources for flowering in fire-prone ecosystems. In low-resource environments, the loss of plant tissue and the allocation of resources for plant defenses are thought to be very costly, because the resources are not easily obtained from the environment [33], especially by slow-growing plants [34] such as the Cerrado’s plants [35, 36]. As a result, the removal of plant tissues by fire or other less intense damages (i.e., the injury imposed by biotic or abiotic stressors eliciting a response from the plant to compensate or tolerate the injury, but not necessarily having a negative impact in the individual) imposes a resource limitation eliciting compensatory physiological responses related to resource allocation to regrowth or reproduction. Fire might act as an analogous consumer of plant tissue affecting the structure and function of fire-prone communities similar to but more intense than leaf removal [37]. Due to these similarities, possibly the compensatory response to damages by fire and by other means such as leaf removal in low-resource environments should be convergent since both impose constraints to plant development.

For a perennial herb, the cost of full recovery of vegetative parts should increase with the amount of damage inflicted to the plant while the cost of reproduction should not change as much for a mature plant. Therefore, the rapid flowering response to fire might not necessarily be an adaptation only to fire or dependency on fire to flower because the apparent increase in fitness can be costly in the long term [38, 39]. Instead, flowering might be stimulated by the extent, severity, and frequency of the damage inflicted on the plant [40, 41]. Here, we tested whether flowering is fire-dependent *sensu* Lamont and Downes [7]—i.e., blooming only in the presence of fire, in contrast to a facultative fire-stimulated species that continues flowering indefinitely at a reduced rate—using *Bulbostylis paradoxa* (Spreng.) Lindm. (Cyperaceae) as a model. *Bulbostylis paradoxa* is a widespread and iconic plant in the Neotropics, which exhibits mass flowering after fires in the dry season and is classified as fire-dependent for flowering and fruiting [17, 42, 43]. Specifically, we seek to answer 1) Whether fire and clipping stimulate *B*. *paradoxa* flowering; 2) If so, how long does it take for burnt and leaf-damaged plants to start flowering after damage compared to unburnt individuals? 3) How do seed characteristics and gemination differ between clipped, burned, and undamaged plants? and 4) Is the flowering of *B*. *paradoxa* independent of fire throughout the year?

## Materials and methods

### Studied species

*Bulbostylis paradoxa*is a widely distributed species in the Neotropics (gbif.org), found in diverse open vegetation types including savannas and grasslands, mostly in dry but also in wet soils. In addition to the Cerrado, *B*. *paradoxa* is distributed along transitional areas with other biomes, such as the Amazon and Pantanal (splink.cria.org.br). It is a perennial evergreen herb with thickened rhizomatous stems (caudex), reaching ca. 25 cm in height and 5 cm in diameter.

The leaf is filiform, rosulate, and prostrate around the stem, and the species presents a few to several single, terminal, lanate, and obovate inflorescences. Fruits are small single-seeded achenes that are obovate to pyriform. The pollen and seed are wind dispersed [44]. The caudex is protected by a dense layer of persistent leaf bases [42, 44]. *Bulbostylis paradoxa* produces new sympodial units, which develop together with the mother plant resulting in vegetative reproduction when detached [45].The root system is shallow, and after burning, *B*. *paradoxa* differs from other Cyperaceae by mobilizing its reserve compounds mainly in the apical part [40]. It is unknown how long it takes for seedlings to reach maturity, or to develop a persistent leaf base to protect the caudex during fire events.

### Study site, number of flowering individuals and inflorescence production

The study was conducted in the Área Alfa da Marinha, ~25 km from Brasília, DF, Brazil (16°00’57’’S, 47°55’43’’W). The site is located at 1,200 m above the sea level. The climate is tropical seasonal, with a well-defined dry season from May to September, and a rainy season from October to April when more than 90% of the annual precipitation occurs. Despite the well-defined seasons the transition periods occur in late September to October (transition from the dry to wet season) and in April to May (transition from the wet to dry season). The mean annual rainfall in the region is 1,453 mm, and the mean temperature is 22°C. The soils are shallow well-drained cambisols, poor in nutrients and acidic. The vegetation is mostly *campo sujo* (grassland with scattered shrubs and small trees [46]) dominated by graminoids, forbs, subshrubs, dwarf palms and patches of the shrub *Vellozia squamata* [26]. The area is subjected to wildfires at the end of the dry season and experienced fires in 2015, and 2017 [26].

For the study, we selected three contiguous plots of *campo sujo* that are part of a fire study initiated in 2018. All areas had small patches of outcrops of rocks, similar soil, vegetation structure and composition, and herbaceous layer fine-fuel load of 572 ± 43 g m^–2^. The first plot was protected from fire since 2015 and used as a control (~ 2000 m^2^). The second and third plots (~ 100 x 30 m each) burned in a wildfire in 2017. In the last week of August 2019 (mid dry-season), a head fire (0.4 ± 0.1m/s) set before mid-day burned the second area in the longitudinal direction consuming ~ 93% of the fine fuel. At the time of the burn air temperature, relative humidity and wind speed were 25.0 ± 0.2°C, 50.7 ± 0.6% and 3.3 ± 1.6 m/s. The third plot was used to investigate the effect of leaf removal by clipping (hereafter referred to as clipped). Before the fire, we randomly tagged 70 individuals of *B*. *paradoxa* in each experimental area (fire and clipped) and 35 individuals in the control. In the fire treatment the individuals were scattered all over the sampling area to ensure that they would be burned independently, under different fire behavior (rate of spread, residence time, and intensity). In the control and clipped areas, the tagged individuals were at least 3 m apart and at least 20 m from the firebreaks to avoid any potential variation due to edge effects. Due to these criteria and because the area protected from fire was smaller than the burned areas, there were fewer individuals to be tagged in the control plot. On the day of the prescribed burn, the 70 individuals in the clipped area had all the leaves removed with scissors (except the leaf bases), as in [23] and [1]. As all individuals were independently sampled in each plot, they were burned or clipped individually and subject to different damages, we considered that our sample unit was each individual plant. In all plots, inflorescence counting started on the day after the fire and clipped treatments. Inflorescences were counted daily in all three plots during the first week of bud flushing and then weekly until seed dispersion, spanning a 2-month study period. In a pilot experiment, we observed that the number of inflorescences per individual and the number of flowering individuals did not change after the first post-fire week.

### Seed germination

In each treatment, 20 days after the flower buds appeared, they were covered with nylon bags to avoid seed dispersal. Based on our results (see below) and in a pilot experiment, the bags did not interfere on seed production and differences on seed germination could be attributed to treatment effects. On the 40^th^ day after flowering, we collected the seeds [17] to measure seed mass, germination, mean germination time, and seed viability in all treatments. In the laboratory, we removed the seeds from the fruits. Due to the low number of seeds, there were three replicates per treatment with 50 seeds each. Each replicate (50 seeds each) was weighed (0.001 g), sown on a filter paper in 9-cm Petri dishes, moistened with 5 mL distilled water in all treatments. The filter paper was remoistened when necessary. Germination was monitored for 45 days at room temperature (~25°C) under white light (12 h/12 h), and the germination criterion was the geotropic curvature of the primary root. After 45 days, we tested the ungerminated seeds for viability with a 1% solution of 2,3,5-triphenyl-tetrazolium chloride. We estimated the mean germination time as described in [47] and the final viability was considered the sum of germinated + seeds that stained red in the tetrazolium test.

### Herbarium data collection

As a proxy of our results and to expand our sampling coverage of flowering *B*. *paradoxa* with or without fire, we examined specimen images of *B*. *paradoxa* from herbarium collections (hereafter referred to as herbarium data) in the SpeciesLink database (https://specieslink.net/). We considered only the high-resolution images from herbarium collections due to the easy identification of the fire scars. We retrieved 586 records of *B*. *paradoxa* for Brazil in the Specieslink database, of which 362 are for the Cerrado region. We considered only records located within the Cerrado biome and within the Brazilian territory, representing a total of 209 individuals with flower stalks (S1 Table). Individual specimens in the herbarium sheets were categorized as “without fire scar” if they had brown stems (thickened rhizome) and abundant leaves (i.e., leaves not burned in recent fires). They were compared with specimens with dark (black) stem tissues, evidence of burn scars on the stem, and the remains of burned leaves and leaf sheaths (S1 Table provide the herbarium data and image links for *B*. *paradoxa*). For each image, we recorded the presence or absence of a fire scar, the month of flowering, and counted the number of flowering stalks. For collections with duplicates, we considered the one with the highest number of flower stalks. When an image contained two or more specimens, we counted the number of flowering stalks only for the one having the largest number of flowering stalks.

### Data analyses

To investigate whether the proportion of flowering individuals of *Bulbostylis paradoxa* is influenced by fire or clipping, we fitted a Generalized Linear Model (GLM) with a binomial distribution. Treatment (control, fire, and clipped) was used as the explanatory variable, and the occurrence of flowering individuals (i.e., presence / absence data) per treatment was used as the response variable. To evaluate how the proportion of flowering individuals (response variable) varied over time (sampling weeks) and among treatments (explanatory variables), we fitted a Generalized Mixed Effect Model (GLMM) with a binomial distribution considering also the interaction between the explanatory variables [48, 49]. We used plant identity as a random factor (repeated measures). We used the package lme4 to fit the GLMMs [48]. Using this same procedure, we determined whether the proportion of individuals flowering within distinct class intervals related to the number of inflorescences per plant differed between the fire and clipped treatments. The significance of all GLM models was assessed using an Analysis of Deviance (ANODEV) with a chi-square test and the GLMM models using an F-test [49]. The factor levels were compared using a model contrast analysis using the library multcomp [49, 50]. Finally, the residuals were analyzed for each GLM and GLMM fitted to test the goodness of fit of each model using the package dharma [51].

Following the same approach, we tested whether seed mass [g/(50 seeds)], germination (%), germination time (days), and seed viability (%) differed among burned, clipped and control plants also fitting separated GLMs for each response variable. All GLMs fitted a gamma distribution [49]. Other procedures were done as described above. All analyses were performed using the software R version 3.6.2 [52].

## Results

**Fig 1:**
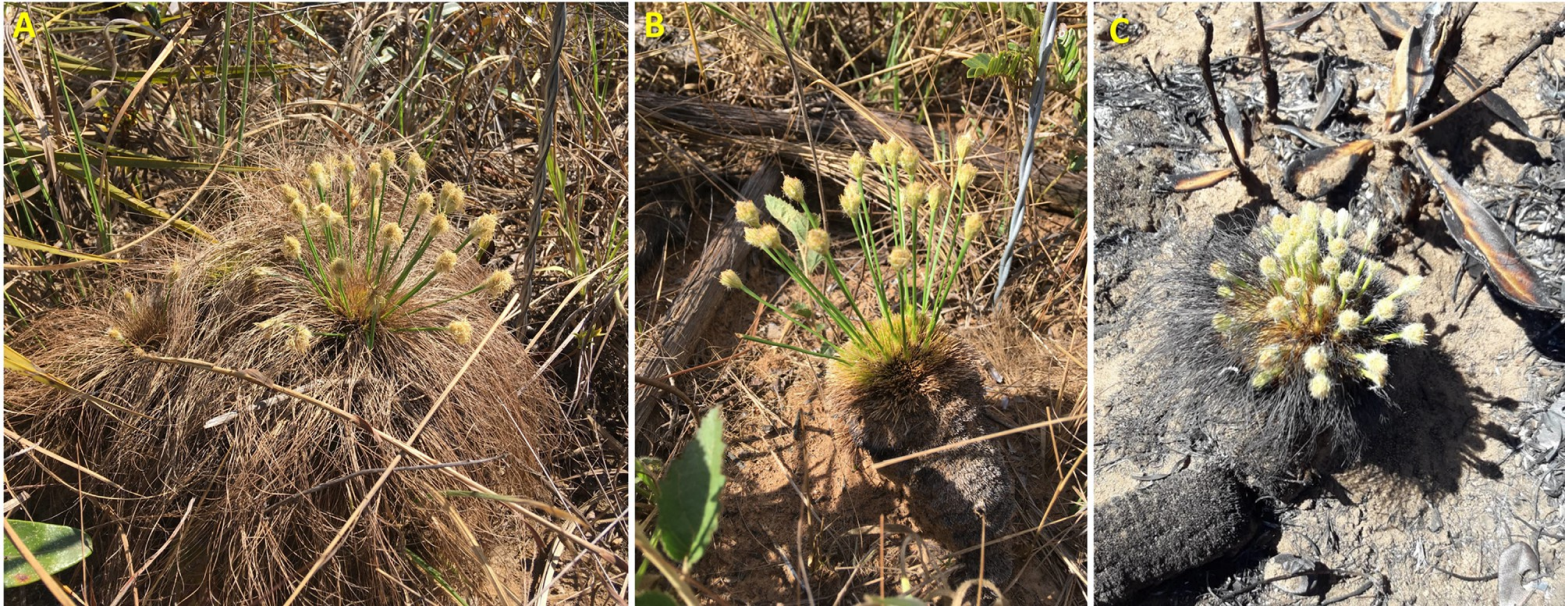
Examples of flowering individuals of *Bulbostylis paradoxa* (Spreng.) Lindm. in the control area (A), after clipping (B), and after a prescribed fire (C) in a *campo sujo* at the Área Alfa da Marinha, Brasília, DF, Brazil. All photos are from individuals of *B*. *paradoxa* observed during the experiment by the authors.

**Fig 2:**
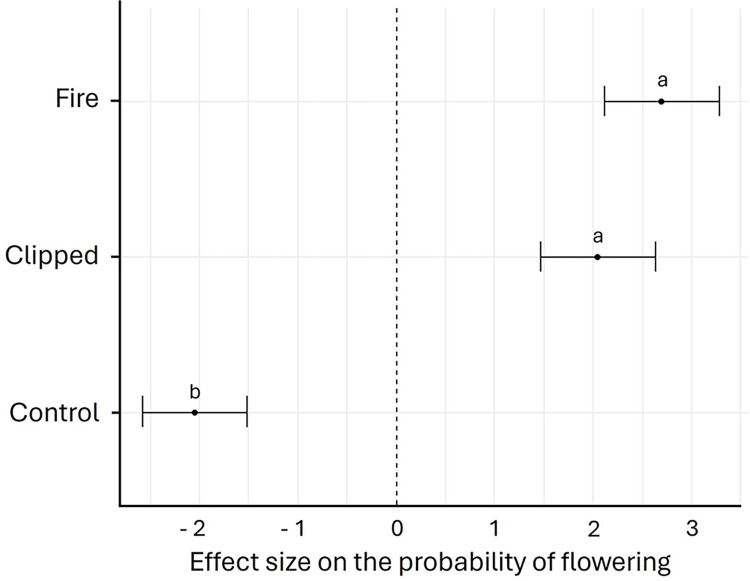
Effect size (with confidence intervals) on the probability of flowering of individuals of *Bulbostylis paradoxa* (Spreng.) Lindm. in the control (control, n = 35), after clipping (clipped n = 70), and after a prescribed fire (fire, n = 70) in a *campo sujo* at the Área Alfa da Marinha, Brasília, DF, Brazil. The effect size refers to the expected probability of flowering individuals irrespective of the time taken for flowering in each treatment based on a Generalized Linear Model (GLM) with a binomial distribution. Different letters above the bars indicate significant differences (model contrast analysis, *P*< 0.05).

**Fig 3:**
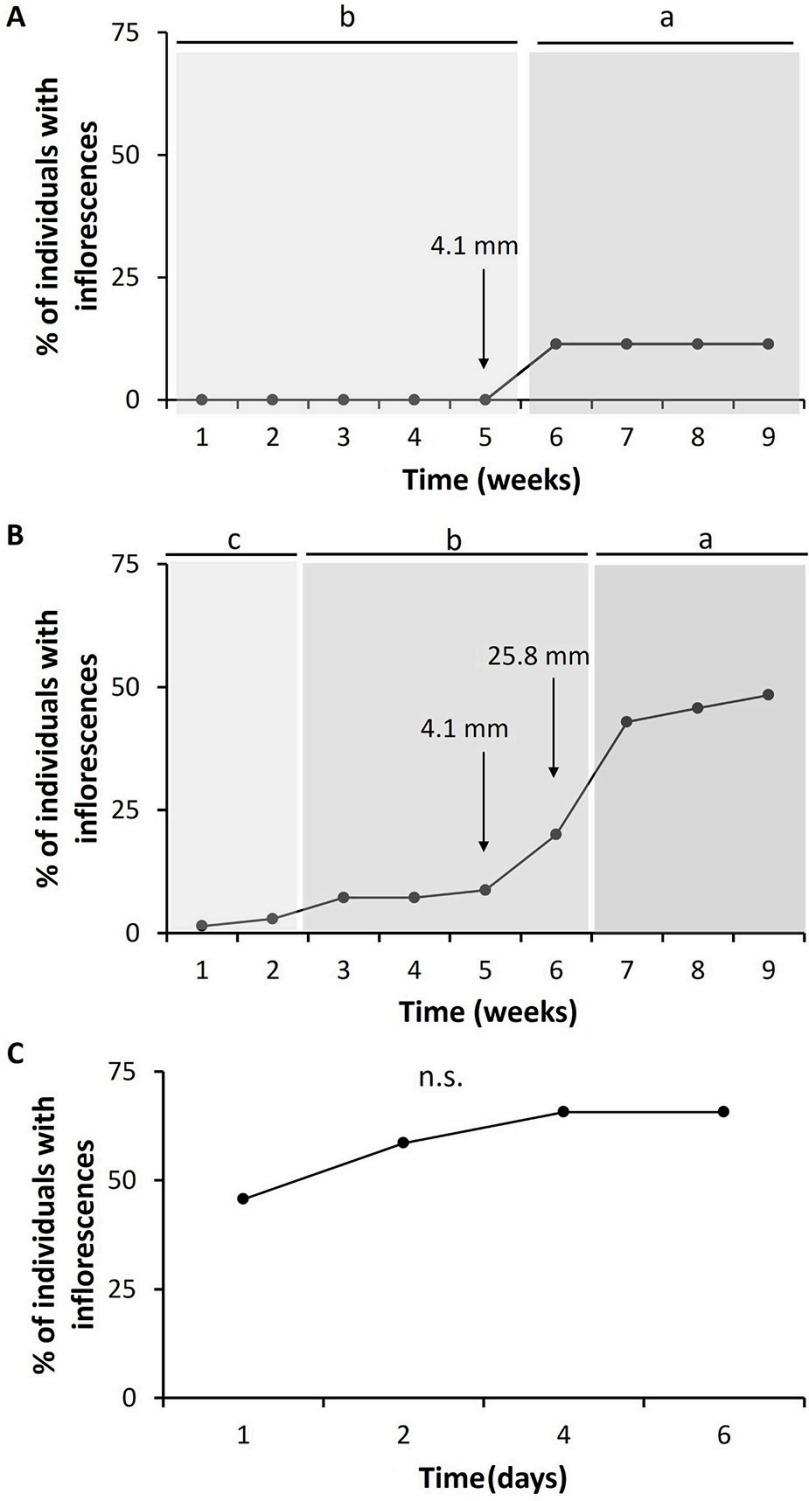
Percentage of flowering individuals of *Bulbostylis paradoxa* (Spreng.) Lindm. over time in the control area (A, n = 35), after leaf clipping (B, n = 70), and after a prescribed fire (C, n = 70), in a *campo sujo* at the Área Alfa da Marinha, Brasília, DF, Brazil. Arrows indicate the amount (mm) of the first rains registered in the area at the onset of the rainy season. Different letters at the top of the graphs indicate significant differences in the proportion of flowering individuals between the time periods (model contrast analysis, *P*< 0.05) and n.s. indicates no significative differences. Note that the scales of the x axes differ among treatments, although the comparison among treatments considered the same scale (weeks). The individuals from all treatments were monitored daily during the first week after the fire and weekly after that until nine weeks after the fire. The number of individuals flowering in the fire treatment stabilized in the first week after the fire. Clipping was performed in the same day of the fire.

**Fig 4:**
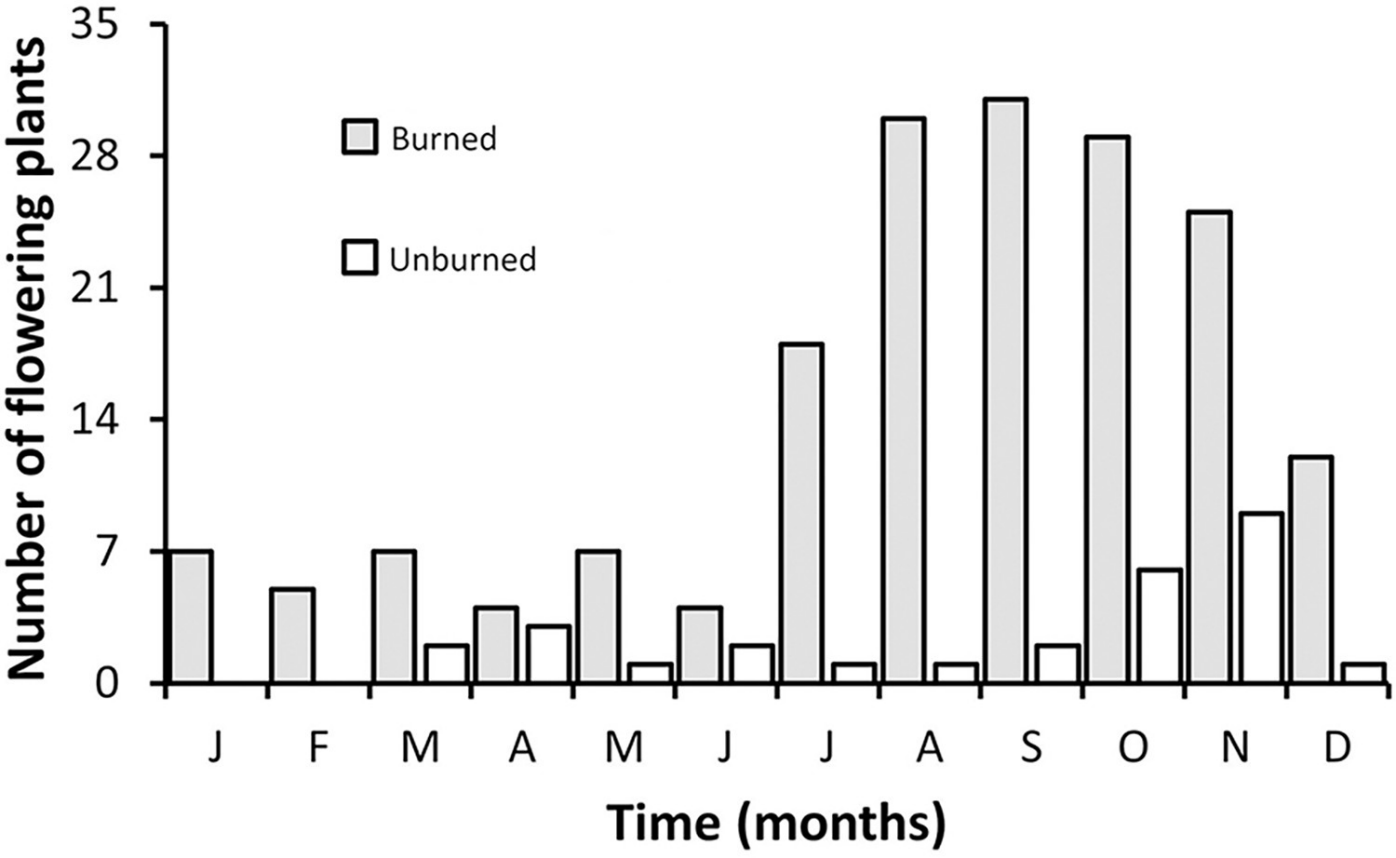
Total number of flowering individuals of *Bulbostylis paradoxa* (Spreng.) Lindm. with (burned) or without (unburned) fire scars through the year in different herbariums that provided high-resolution images in the SpeciesLink database for the Cerrado biome (n = 209).

*Bulbostylis paradoxa* flowered in all treatments (Fig 1). However, the probability of flowering was different among the treatments (χ^2^ = 30.53, d.f. = 2, *P*< 0.001). The proportion of flowering individuals was highest in the fire (65.7%) and clipping (48.6%) that although did not differ from each other, differed from the control treatment (11.4%) (Fig 2). Considering time for flowering, we found that the proportion of individuals flowering differed among treatments (F = 13.10, d.f. = 2, *P* < 0.001), through time (F = 16.78, d.f. = 7, *P* < 0.001), and there was an interaction between treatment and time (F = 11.84, d.f. = 2, *P* < 0.001). After the fire, production of floral buds was observed within 24 hours, after clipping within the first week, and in the control treatment only during the 5th week. Flowering stalks were fully elongated two weeks after blooming in all treatments. Significantly more individuals flowered more readily in the fire treatment than in all other treatments, accordingly the model contrast analysis (*P* < 0.05). However, a significantly higher proportion of clipped individuals flowered more readily than the control group, suggesting that damage anticipate flowering (Fig 3). In the control the maximum number of flowering individuals occurred after the 5th week and after a 4.1 mm rainfall (Fig 3A), and for the clipped plants, the maximum number of inflorescences was observed after the 7th week, preceded by 2 days of rain (Fig 3B). However, in the fire treatment most individuals flowered within six days after burning (Fig 3C). The individuals flowered more readily after the fire than in the other treatments, but after the 4th day, the number of burnt individuals with inflorescences stabilized (Fig 3C). Nevertheless, for the clipped plants ca. 10% of the individuals already flowered before the 7th week, and there were three distinct flowering periods before reaching peak bloom (Fig 3B). For the herbarium data, we found 85.7% of the sheets with burned plants, and 14.3% were not burned. Flowering unburned individuals occurred mainly in the wet season (October-April) and transition from the dry to wet season (end of September-October), while flowering burned individuals in the dry season (May-September) and at the beginning of the wet season (October-November) (Fig 4).

**Fig 5:**
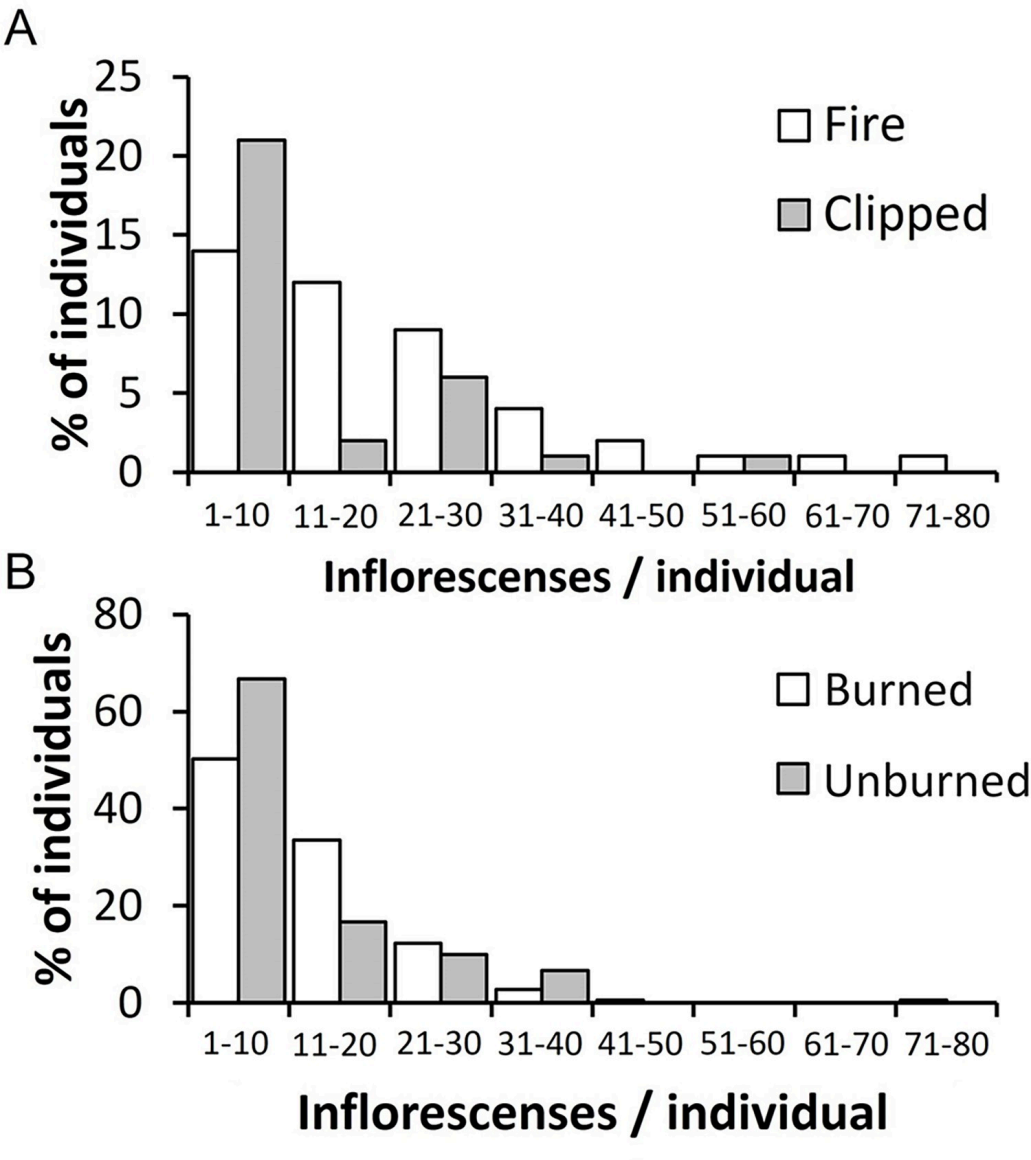
A) Percentage of *Bulbostylis paradoxa* (Spreng.) Lindm. individuals with different numbers of inflorescences per individual after a prescribed fire (Fire, n = 70), and after leaf removal (Clipped, n = 70) in a *campo sujo* in the Área Alfa da Marinha, Brasília, DF, Brazil. Data for the control area is not included (n = 35, however, only four with inflorescences). B) Percentage of *B*. *pardoxa* with different number of flowering stalks with (burned) or without (unburned) fire scars in the high-resolution images of the SpeciesLink database for the Cerrado biome (n = 209).

Overall, the mean number of inflorescences per individual was 12 ± 9 in control (n = 4), 10 ± 2 (n = 34) in clipped, and 22 ± 3 in fire (n = 46) treatments. From the herbarium data, the mean number of flowering stalks/herbarium sheet was 10.4± 8.6 (n = 30) for the unburned and 12.8± 9.8 (n = 179) burned individuals. The maximum inflorescences per individual were 39 in control, 51 in clipped, and 79 in the fire treatments, contrasting with 30 and 80 for unburned and burned plants in the herbarium data. However, individuals with 1–10 inflorescences represented 75% in control, 30% in fire, and 62% in the clipped treatments. In the herbarium data 66.7% unburned individuals and 39% of burned had 1–10 flowering stalks. Individuals with more than 30 inflorescences represented 3% in control, 1% in the clipped, and 21% in fire treatment, while 1% for unburned individuals and 4% of burned from the herbarium data. The mean number of inflorescences per individual did not differ significantly between fire and clipped treatments (χ^2^ = 0.29, d.f. = 14, *P* = 0.591) (Fig 5).

**Fig 6:**
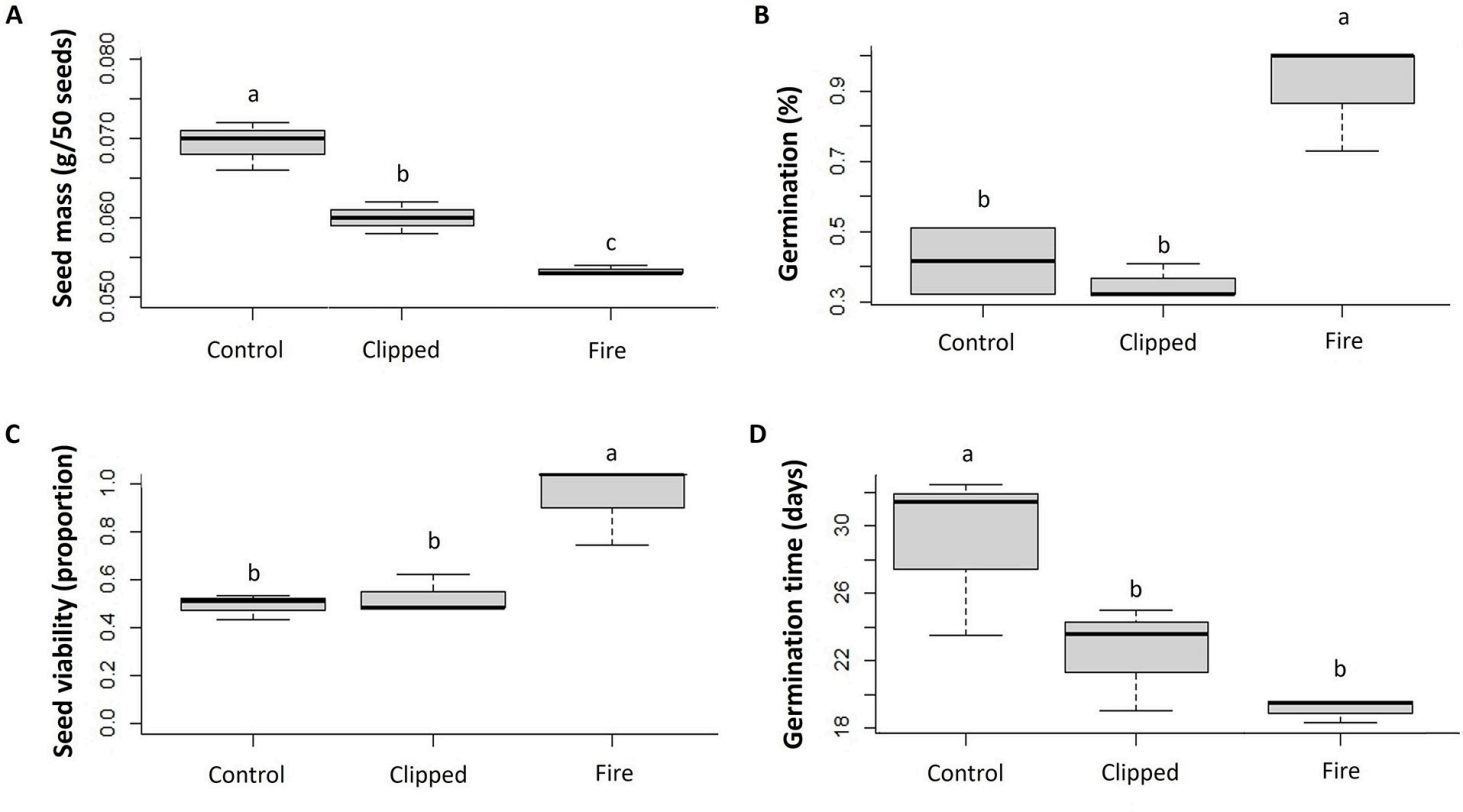
Germinative parameters (± SE) of *Bulbostylis paradoxa* (Spreng.) Lindm. in the control area (Control), after a prescribed fire (Fire), and after leaf removal (Clipped), considering plant seed mass (A), seed germination (B), seed viability (C) and germination time (D) in a *campo sujo* at the Área Alfa da Marinha, Brasília, DF, Brazil. Different letters at the top of each bar indicate significant differences in mean values (model contrast analysis, *P*< 0.05). Seed viability = germinated + those stained red with tetrazolium after 45 days.

Seed mass was higher in the control treatment and smaller for burned individuals (χ^2^ = 42.54, d.f. = 2, P = 0.0003; Fig 6A). Seeds of burned plants had about 4-fold higher percent seed germination (61.3± 8.7%) than the control ones (15.3 ± 4.4%; Fig 6B), although control and clipped treatments did not differ from each other. However, seed viability (χ^2^ = 14.13, d.f. = 2, P = 0.0054) was also low in the control plants and in the clipped plants compared to burned plants (Fig 6C). Following the opposite trend, the germination time was 10 days longer in the control than in the other treatments (χ^2^ = 2.64, d.f. = 2, P = 0.0005) (Fig 6D).

## Discussion

We showed that *B*. *paradoxa* is not a strictly fire-dependent species as previously reported [17, 42, 43], but rather, it is fire-stimulated flowering species (*sensu* [7]) since flowering was also observed in several clipped plants and in a small percentage of undamaged and unburned individuals (Figs 1–3). The damage imposed by fire resulted in a higher investment in early reproduction and flowering synchronism in line with the reported for the species in the literature. For the unburned area, the small number of flowering individuals agrees with the results retrieved from the herbarium data; 14.3% of the images were from individuals flowering without fire scars, especially in the rainy season. Both findings are in line with the low percentage of flowering individuals reported in undisturbed Cerrado herbaceous layer, either at community [15, 16, 21] or population level [53]: 3% to 19%. Damage rather than nutrient turnover may explain burned and clipped individuals flowering in our experiment because during the time we carried out the experiment (the end of the dry season) there were more than 100 days without rain. In these conditions it is likely that nutrients are immobilized above ground as dry biomass or as ashes, in the case of burned areas.

As in other ecosystems, both fire and removal of aerial biomass in herbaceous species by different means (total pruning, total defoliation, or removal of apices) greatly increase flower production of the Cerrado species [1, 3, 20, 21]. Recent studies have shown that even a small amount of leaf damage may induce a significant increase in flowering [54], and the removal of the dry leaves (except for the leaf base) also stimulated flowering of *B*. *paraxoda* similar to the recently reported for *Rhynchospora confusa* (Cyperaceae) [55]. *Bulbostylis paradoxa* has a shallow root system, and does not have a prostrate architecture suggesting that the species did not evolve under intense grazer pressure [56]. Nevertheless, vegetative propagation of *B*. *paradoxa* is ensured by the detachment of the sympodial units from the mother-plant after damage [45], which might also stimulate flowering.

The caudex of *B*. *paradoxa* is protected from the heat pulse during fires by a dense layer of persistent leaf bases that insulate their vascular and meristematic tissues [42, 44], and blooming initiates within the first 24 hours after a fire [17]. Fire-induced flowering of *B*. *paradoxa* may occur at any time of the dry season [28] and, as suggested by our herbarium data, independently of fire season. Clipped individuals initiated blooming on the 7th day after the damage, and by the third week, 10% of the plants flowered. Similarly, Haddad and Válio [1] report a delay between flowering in burned individuals of *Lantana montevidensis* and those that underwent a complete pruning. Smoke compounds, mostly ethylene, could also stimulate post-fire flowering [6, 10]. Although smoke can drift to unburned areas and stimulate the flowering of unburned individuals [57, 58], this was not corroborated for *B*. *paradoxa* by Fidelis et al. [17] nor for the undamaged plants in our study. On the other hand, clipping as a fire stimulus and anticipation of flowering have been associated with the amount of vegetative part removed, the increased light availability, and with the season of leaf removal [1, 3, 5, 6, 59, 60].

To our knowledge, the mechanism that stimulates *B*. *paradoxa* flowering is still unknown, especially after a long dry season burn (i.e., more than 100 days without rain). A significant increase in the flowering of undamaged and clipped individuals occurred after precipitation of 4.1 mm, and the maximum number of individuals flowering was time dependent and associated with the onset of the rainy season (Fig 3). The blooming of individuals in the control plot might be a response to the first rains that alleviate the water deficit period imposed by the long dry season. However, there is a delayed response in comparison to burned plants. The reconstruction of the flowering period of unburned *B*. *paradoxa* for the Cerrado region (Fig 5) also confirms that, a small number of unburned *B*. *paradoxa* flowers during the dry season with a significant increase of flowering plants at the beginning of the rainy season (late September/October), as for other species of Cerrado’s herbaceous layer [15, 29].

Although not fire-dependent, the fire stimuli resulted in more individuals flowering, more inflorescences, more seeds with higher viability (2.5-fold) and germination (4-fold), and lower germination time than in the control area. This would be advantageous under the natural fire regime of Cerrado, with fires occurring during the rainy season and in the transition between dry to rainy seasons when the seeds will be dispersed in a system without the competition for light and space and enriched by the deposition of ash and smoke compounds that become available with the rains [18, 19]. These post-fire conditions could be beneficial for seedling establishment. However, the few studies on the smoke and ash effects on the germination of Cerrado seeds show, in general, that smoke or ash alone does not stimulate germination [43, 61–64]. Nevertheless, due to the fast recovery of the herbaceous layer biomass after the fire, mostly resprouts from underground organs [53], in 6 months the proportion of bare soil might decrease to circa 20% [53, 65], increasing competition for light. During this short period, the new seedlings must develop a deep enough root system to survive the long period of severe soil water deficit of the next dry season [66] and a leaf base protection to insulate the vascular tissues to survive an eventual fire episode. However, to the moment, it is unknown if an efficient leaf base protection can be constructed in the first growing season. As *B*. *paradoxa* produces non-dormant seeds [64], after fires in the dry-wet season transition the time between blooming, stalks elongation, seed maturation, and dispersion may allow germination to occur when rainfall is more constant, thus avoiding root desiccation of the newly emerged seedling during the short dry spells at the onset of the rains.

Time between blooming and seed dispersal will also benefit the establishment of seedlings of *B*. *paradoxa* in unburned areas. Our results suggest that even a minor amount of rain stimulate synchronous flowering of unburned plants at dry-wet season transition. The time elapsed between blooming and seed maturation will allow seeds to be dispersed in an environment enriched by the nutrients released during litter decomposition [67, 68]. Although water availability will not limit germination, the seeds will depend on safe sites to germinate since the occurrence of bare soil is low, ~ 10% to 20% [53, 65], and competition for light below the herbaceous layer is high [69]. Although the number of individuals blooming, number of inflorescences, seed viability, and germination are lower than in burned sites, the individuals recruited via seeds, added to vegetative reproduction [45] may ensure site occupation, genetic variability, and population persistence in unburned areas.

Plant species are not necessarily adapted to fire but to a fire regime that includes season, frequency, and burned area [9, 18]. Natural fires in the open physiognomies of Cerrado are initiated by lighting during the dry-wet season transition, usually followed by rain, or in the short dry spells at the onset of the rainy season (5 to 10 days), with a frequency of 1 to 9 years [70]. While burned areas in the dry-wet season transition can reach >10000 ha, during the short dry spells in the rainy season the burned area is smaller than 500 ha [70, 71]. Nowadays fire regime has changed, and most Cerrado fires are anthropogenic, burning large areas in the dry season (May to September), with a frequency varying from 1 to 4 years [70, 72]. As *B*. *paradoxa* flowers at any time after a dry season burn, after fires early in the dry season (late May to June), the seeds will be dispersed in an increasingly harsh environment, remaining in the dry soil from three to five months until the onset of the rainy season when germination starts [64]. Then, when the rains come, the newly germinated seeds and seedlings resulting from synchronous early flowering would then be exposed to the dry spells that typically occur at this time of the year, increasing seedling mortality. The ash and smoke compounds may not be available for germination and seedling development at the onset of the next rainy season since they are short-lived [20, 73]. Moreover, ash deposition on the soil surface and removal of vegetation cover increases daily soil temperature amplitudes by as much as 35°C, which might reduce seed germination and viability [74, 75]. Furthermore, as emergence is constrained by seed mass, seedlings from light-weight seeds such as those *B*. *paradoxa* may not be able to push through the soil column if they are deeply buried in the seed bank [76, 77]. Additionally, a significant seed loss due to ant removal may increase in burned areas and last up to the recovery of herbaceous vegetation [78–80] and might result in a significant loss of seeds. Regarding alteration in fire frequency, *B*. *paradoxa* might not recover the reserves invested in such a massive production of flowers and seeds if fire frequency is increased [40] and seedling survival to high fire frequency (1 to 4 years) remains to be investigated.

## Conclusions

We showed that *B*. *paradoxa* is not a fire-dependent species since fire and clipping anticipated the flowering season and highly increased the number of flowering plants and seeds when compared with undamaged ones. Even so, intermittent flowering and propagation by fragmentation of *B*. *paradoxa* plants in unburned areas can ensure genetic variability, population persistence, and site occupancy. The observed stimulation of flowering by fire is an adaptive response to damage of the aerial parts that is modulated by the amount of damage inflicted to the plant as shown by Coutinho [23] and Haddad and Válio [1] for *Lantana montevidensis* and by Pilon et al. [55] for *R*. *confusa*, Cerrado`s herbaceous species, or for plant communities as reported by Araújo et al. [21]. Yet, as the fire-stimulated flowering is independent of fire season, the changes in fire regime imposed by anthropogenic fires might significantly impact the benefits of such adaptation. Therefore, we propose that plant damage by fire or another agent might also trigger, in other herbaceous layer species, a multi-response process, as recently reported by Pilon et al. [55] which observed a single individual of *R*. *confusa* half-mowed flowering in an unburned area. Classifying a species as fire-dependent for flowering requires experimental data and detailed phenological studies, that evaluate the effect of multiple stressors on flowering and on vegetative reproduction and propagation by fragmentation. Also, reports of fire-dependent flowering in the Cerrado should be interpreted with caution since flower stimulation is a strategy for species persistence that evolved before the current constant and severe fire episodes in the biome, that nowadays are mainly due to unnatural causes.

## Supporting information

S1 Table *Bulbostylis paradoxa* herbarium sheets with images examined in the SpeciesLink database (splink.cria.org.br) to search the occurrence of fertile specimens with and without occurrence of fire scars in the collections. (XLSX)
